# CYP1A2 mRNA Expression Rather than Genetic Variants Indicate Hepatic CYP1A2 Activity

**DOI:** 10.3390/pharmaceutics14030532

**Published:** 2022-02-27

**Authors:** Ferenc Fekete, Katalin Mangó, Annamária Minus, Katalin Tóth, Katalin Monostory

**Affiliations:** 1 Institute of Enzymology, Research Centre for Natural Sciences, Magyar Tudósok 2, H-1117 Budapest, Hungary; fekete.ferenc@ttk.hu (F.F.); mango.katalin@ttk.hu (K.M.); minus.annamaria@ttk.hu (A.M.); tubakati@gmail.com (K.T.); 2 Doctoral School of Biology and Institute of Biology, Eötvös Loránd University, Pázmány Péter Sétány 1/A, H-1117 Budapest, Hungary

**Keywords:** CYP1A2, phenacetin *O*-dealkylation activity, CYP1A2 expression, *CYP1A2* genetic polymorphisms, non-genetic factors, smoking, chronic alcohol consumption, amoxicillin+clavulanic acid, ciprofloxacin, carbamazepine

## Abstract

CYP1A2, one of the most abundant hepatic cytochrome P450 enzymes, is involved in metabolism of several drugs and carcinogenic compounds. Data on the significance of *CYP1A2* genetic polymorphisms in enzyme activity are highly inconsistent; therefore, the impact of *CYP1A2* genetic variants (−3860G>A, −2467delT, −739T>G, −163C>A, 2159G>A) on mRNA expression and phenacetin *O*-dealkylation selective for CYP1A2 was investigated in human liver tissues and in psychiatric patients belonging to Caucasian populations. CYP1A2*1F, considered to be associated with high CYP1A2 inducibility, is generally identified by the presence of −163C>A polymorphism; however, we demonstrated that −163C>A existed in several haplotypes (CYP1A2*1F, CYP1A2*1L, CYP1A2*1M, CYP1A2*1V, CYP1A2*1W), and consequently, CYP1A2*1F was a much rarer allelic variant (0.4%) than reported in Caucasian populations. Of note, −163C>A polymorphism was found to result in an increase of neither mRNA nor the activity of CYP1A2. Moreover, hepatic CYP1A2 activity was associated with hepatic or leukocyte mRNA expression rather than genetic polymorphisms of *CYP1A2*. Consideration of non-genetic phenoconverting factors (co-medication with CYP1A2-specific inhibitors/inducers, tobacco smoking and non-specific factors, including amoxicillin+clavulanic acid therapy or chronic alcohol consumption) did not much improve genotype–phenotype estimation. In conclusion, *CYP1A2*-genotyping is inappropriate for the prediction of CYP1A2 function; however, CYP1A2 mRNA expression in leukocytes can inform about patients’ CYP1A2-metabolizing capacity.

## 1. Introduction

CYP1A2, one of the most abundant drug-metabolizing cytochrome P450 (CYP) enzymes in the human liver, is involved in phase I biotransformation processes. Its relative amount in hepatic microsomes is approximately 13–15% of the total CYP pool [1], and it is scarcely expressed in extrahepatic tissues. Sporadic reports have, however, cited CYP1A2 expression in leukocytes, the colon and rectum [2,3] as well as in the lung, where CYP1A2 mRNA and enzyme protein have been detected at a low level [4]. CYP1A2 catalyzes the metabolism of several clinically important drugs (e.g., olanzapine, clozapine, duloxetine, lidocaine, theophylline, caffeine and tacrine) and endogenous molecules (e.g., retinoids, 17β-estradiol, estrone, melatonin, uroporphyrinogen and arachidonic acid). It is also involved in the bioactivation of procarcinogenic compounds (e.g., polycyclic aromatic hydrocarbons and heterocyclic aromatic amines) and mycotoxins (e.g., aflatoxin B1) [5,6].

The *CYP1A2* gene is located on chromosome 15 in a head-to-head orientation with *CYP1A1* and consists of seven exons and six introns with a non-coding region in exon 1. The regulatory region of *CYP1A2* overlaps with that of the *CYP1A1* gene; therefore, some common characteristics have been recognized in transcriptional regulation of *CYP1A2* and *CYP1A1* [7,8,9]. Both the CYP1A2 and CYP1A1 enzymes are highly inducible, and the common promoter region contains several copies of regulatory elements and transcription factor binding sites, such as the xenobiotic responsive element (XRE) [6,10]. Activation of the aromatic hydrocarbon receptor (AhR) by xenobiotics (e.g., omeprazole, aromatic hydrocarbons, components of tobacco smoke and charcoaled meat) is involved in transcriptional induction of *CYP1A* genes [11]. The ligand-activated AhR forms a heterodimer with the AhR nuclear translocator (ARNT), and this transcriptional activator complex interacts with the XRE in the regulatory region to initiate transcription of *CYP1A* genes. Furthermore, several endogenous factors, both genetic and non-genetic, have been reported to contribute to the large inter-individual differences observed in CYP1A2 mRNA expression and activity [1,11,12,13].

The variability in CYP1A2 activity is often explained by *CYP1A2* genetic polymorphisms. *CYP1A2* allelic variants contain one or more single nucleotide polymorphisms (SNPs), and, further, a particular SNP can be present in several haplotypes, which makes the identification of allelic variants laborious [14]. To date, more than 40 *CYP1A2* alleles and haplotypes have been listed by PharmVar (Pharmacogene Variation Consortium), which attributes increased inducibility or decreased expression to several of these alleles (https://www.pharmvar.org/gene/CYP1A2, access date: 12 January 2022). The most frequently studied *CYP1A2* SNP is the −163C>A nucleotide change (rs762551) in intron 1, generally designated as CYP1A2*1F. CYP1A2*1F has been suggested to be associated with increased inducibility observed in smokers or in subjects treated with the CYP1A2 inducer omeprazole; however, the contribution of −163C>A to CYP1A2 activity was recognized as inconsistent in the literature [15,16,17,18,19,20,21]. Most studies considered CYP1A2*1F to be identical to −163C>A; however, −163C>A is also part of other haplotypes (CYP1A2*1J, CYP1A2*1K, CYP1A2*1L, CYP1A2*1M, CYP1A2*1V and CYP1A2*1W) [22,23]. Due to the strong linkage disequilibrium between various *CYP1A2* polymorphisms, such as −163C>A (rs762551), −3860G>A (rs2069514), −2467delT (rs35694136), −739T>G (rs20695) and 2159G>A (rs2472304), the functional relevance of a particular SNP in CYP1A2 activity may differ from that of the haplotype(s). The −3860G>A (rs2069514) polymorphism identified in CYP1A2*1C has been reported to be associated with decreased metabolism of the CYP1A2 probe substrate caffeine [16,24], and, similarly, the decreased CYP1A2 activity was attributed to the −2467delT (rs35694136) polymorphism in CYP1A2*1D [16]. However, the increased CYP1A2 inducibility in smokers carrying −163C>A appeared to be reversed in those subjects with CYP1A2*1L, where −163C>A exists in linkage disequilibrium with −3860G>A and −2467delT [16,20]. Furthermore, the increased inducibility due to the −163C>A polymorphism has been demonstrated to be abolished by the linkage disequilibrium with −2467delT in subjects that carry CYP1A2*1V or CYP1A2*1W [20]. The −163C>A polymorphism often occurs in linkage disequilibrium with 2159G>A (rs2472304) in CYP1A2*1M; however, the 2159G>A nucleotide change has been reported to have no effect on caffeine metabolism [25]. CYP1A2*1K, which is identified by the haplotype with three polymorphisms, −163C>A (rs762551), −739T>G (rs20695) and −729C>T (rs12720461), has been reported to display reduced CYP1A2 activity, which was primarily attributed to the −729C>T polymorphism affecting a putative transcription factor binding site [26]. In addition to *CYP1A2* genetic variability, significant interethnic variation in the *CYP1A2* allele and haplotype frequencies has been demonstrated [14], and, due to misinterpretation of the haplotypes from SNP data, some inconsistency in frequency estimation in various populations was recognized, further complicating the evaluation of phenotype consequences.

Genetic polymorphisms seem to explain only 25–33% of inter-individual variation in CYP1A2 activity and expression, and non-genetic factors (e.g., sex, age, nutrition, diseases, hormonal status, smoking and medication) that regulate *CYP1A2* gene expression or inhibit enzyme function may also contribute to the inter-individual variability of the CYP1A2 phenotype to some extent [10,27,28]. It has been indicated that hepatic CYP1A2 expression and activity was influenced by gender and age as intrinsic non-genetic factors [29,30]; however, a current study did not confirm the sex- and age-related differences in CYP1A2 function [31]. Clear evidence for the AhR-mediated induction of CYP1A2 transcription by omeprazole, components of tobacco smoke or consumption of broccoli and brussels sprout has been provided [20,32,33], whereas indirect mechanisms, which up- and downregulate nuclear receptors and transcription factors, have also been suggested to modify CYP1A2 expression [11]. Furthermore, medication with well-known CYP1A2 inhibitors, such as ciprofloxacin, fluvoxamine and oral contraceptives, has been demonstrated to substantially reduce CYP1A2 activity [34,35,36]. Therefore, investigation of both genetic and non-genetic factors is required for appropriate estimation of CYP1A2-mediated metabolism. The main aim of the present study was to evaluate the influence of *CYP1A2* polymorphisms on CYP1A2 activity and mRNA expression in human liver samples and in patients with psychiatric disorders. The hepatic microsomal CYP1A2 activity was characterized by using phenacetin as the CYP1A2-selective probe substrate [10]. Furthermore, we aimed to identify non-genetic factors (demographic parameters, medication, smoking and alcohol consumption) that can potentially modify CYP1A2 expression and/or activity; thus, a more complex picture was expected to be obtained about the major determinants of CYP1A2 expression and function.

## 2. Materials and Methods

Human liver tissues (N = 151) were obtained from organ transplant donors at the Department of Transplantation and Surgery, Semmelweis University (Budapest, Hungary). Human livers not selected for transplantation or liver tissues remaining after reduced-size transplantation were used in the present study. Furthermore, 274 unrelated inpatients at the Department of Psychiatry and Psychotherapy (Semmelweis University) were involved in the study. All subjects were 18 years of age or older. The demographic data and clinical histories of the liver tissue donors and patients with psychiatric disorders were recorded (Table 1).

### 2.1. Human Liver Microsomes and RNA Samples

Human liver tissues were perfused with Euro-Collins solution (Fresenius AG, Bad Homburg vdH, Germany) and excised. For isolation of the microsomal fraction, the tissues were homogenized in 0.1 M Tris-HCl buffer (pH 7.4) containing 1 mM EDTA and 154 mM KCl, and differential centrifugation was performed as described by van der Hoeven and Coon [37]. Microsomal protein content was determined by the method of Lowry et al. using bovine serum albumin as the standard [38]. Total RNA was extracted from the liver tissues (approximately 50 mg), and from leukocytes (approximately 10^7^ cells) isolated from peripheral blood samples using TRIzol reagent (Invitrogen, Carlsbad, CA, USA), according to the manufacturer’s instructions. The RNA samples were stored in ultra-pure water containing 0.1% diethylpyrocarbonate at −80 °C for further analyses.

### 2.2. CYP1A2 Enzyme Assay

The method of Butler et al. was followed to determine the hepatic phenacetin *O*-dealkylation activity selective for CYP1A2 [39]. The incubation mixture contained a NADPH-generating system (1 mM NADP, 10 mM glucose 6-phosphate, 5 mM MgCl_2_ and 2 units/mL glucose 6-phosphate dehydrogenase), human liver microsomes (1 mg/mL) and phenacetin (200 μM). After a 20-min incubation period, the reaction was terminated by ice-cold methanol, and the incubation mixture was centrifuged for 10 min at 10,000× *g*. The metabolite (acetaminophen) formation was determined by a high-performance liquid chromatographic analysis, using Inertsil ODS-4 column (75 × 2.1 mm, 3 μm; GL Sciences Inc., Tokyo, Japan) and a mobile phase containing 30% acetonitrile and 0.1% formic acid [39]. A CYP1A2 enzyme assay for each donor was performed in triplicate, and the activity was expressed as pmol acetaminophen/(mg protein ∗ min).

### 2.3. CYP1A2 Genotyping

Genomic DNA was isolated from the liver tissues and peripheral blood samples by Quick-DNA™ Universal Kit (Zymo Research, Irvine, CA, USA). Hydrolysis SNP analyses for −3860G>A (rs2069514), −2467delT (rs35694136), −739T>G (rs206926), −163C>A (rs762551) and 2159G>A (rs2472304) were performed by a polymerase chain reaction (PCR) with commercially available (Thermo Fisher Scientific, Waltham, MA, USA) or self-designed TaqMan assays (Supplementary Table S1). Real-time PCR was carried out with 30 ng of genomic DNA by using Luminaris Color Probe qPCR Master Mix (Thermo Fisher Scientific). The incubation protocol was as follows: 50 °C for 2 min, 95 °C for 10 min and 35 cycles of 95 °C for 15 s, 61 °C for 1 min.

### 2.4. Analysis of CYP1A2 mRNA Levels by Quantitative Real-Time PCR

Total RNA (3 μg) was reverse-transcribed into single-stranded cDNA by using Maxima First Strand cDNA Synthesis Kit (Thermo Fisher Scientific, Waltham, MA, USA). The real-time PCR with human cDNA was performed by using KAPA Fast Probes Mastermix (KAPA Biosystems, Cape Town, South Africa) and TaqMan probes for CYP1A2 (Bio-Search Technologies, Novato, CA, USA). The incubation protocol was 95 °C for 3 min and 45 cycles of 95 °C for 3 s, 58 °C for 30 s. The quantity of the target mRNA relative to that of the housekeeping gene glyceraldehyde 3-phosphate dehydrogenase (*GAPDH*) was determined. GAPDH expression was set to 1, and CYP1A2 mRNA levels were normalized by the GAPDH expression. The sequences of primers and probes used for the real-time PCR analyses of CYP1A2 and the GAPDH expression were previously reported by Déri et al. [40].

### 2.5. Statistical Analysis

For liver tissue donors (N = 151) and psychiatric patients (N = 274), *CYP1A2* genotypes (for CYP1A2*1C, CYP1A2*1D, CYP1A2*1E, CYP1A2*1F, CYP1A2*1L, CYP1A2*1M, CYP1A2*1V and CYP1A2*1W) were determined. Hepatic CYP1A2 activities (N = 131) and/or the mRNA expression (N = 93) of CYP1A2, as well as the CYP1A2 expression in leukocytes (tissue donors N = 35; psychiatric patients N = 274) were also determined. For reconstructing the *CYP1A2* haplotypes from the SNP data based on a Markov chain Monte Carlo algorithm, we used PHASE software (v2.1; Department of Statistics, University of Washington, Seattle) [41,42]. The frequency distribution of CYP1A2 activities was determined in 131 liver tissue donors, and three categories (low, intermediate, high) were distinguished for poor, intermediate and normal metabolizers. The comparison of CYP1A2 enzyme activities or mRNA levels between various groups (*CYP1A2* genotype or phenotype groups or even gender-age groups) was performed by Kruskal–Wallis ANOVA followed by Dunn’s multiple comparisons test (GraphPad Instat v3.05; GraphPad Software, San Diego, CA, USA). Phenoconversion frequencies were evaluated on the basis of the non-genetic factor frequencies (medications, chronic alcohol consumption and smoking) in the medical histories of tissue donors in various genotype-based phenotype groups by Fisher’s exact test. Linear regression models (Supplementary Table S2) were formulated to test potential associations between CYP1A2 activity (N = 131) or mRNA expression (N = 93) as dependent variables and *CYP1A2* SNPs, haplotypes, sex, age (under 50 and above 50 years old), chronic alcohol consumption and medication with amoxicillin+clavulanic acid as covariates. Multiple linear regression analyses were carried out by IBM SPSS Statistics software [v28.0.1.0 (142), IBM Corp., Armonk, NY, USA]. A *p* value of < 0.05 was considered to be statistically significant.

## 3. Results

### 3.1. CYP1A2 Haplotypes and Genotypes of Liver Tissue Donors and Psychiatric Patients

The *CYP1A2* SNPs most common in Caucasian populations (−3860G>A, −2467delT, −739T>G, −163C>A and 2159G>A) were identified in 151 liver tissue donors and in 274 patients with psychiatric disorders. Those subjects who did not carry any of the investigated polymorphisms were considered to have the CYP1A2*1 wild-type allele. The relative frequencies of the *CYP1A2* haplotypes and genotypes in the liver tissue donors and patients with psychiatric disorders were compared to those reported in Caucasian populations (Table 2) [20,25,43,44]. The frequency of the wild-type CYP1A2*1, often designated as CYP1A2*1A (32.8% and 32.1%), was similar to that was reported in Caucasians [20,25,43]. Although the liver tissue donors and psychiatric patients all belonged to Caucasian populations, the frequencies of several *CYP1A2* haplotypes markedly differed from that of the previously reported frequency data in Caucasians. The CYP1A2*1C, CYP1A2*1D and CYP1A2*1E alleles were identified in 0.4–4%, 3.4–11% and 0.4–6% of the Caucasian populations [44]; however, these alleles were detected neither in the tissue donors nor in the patients with psychiatric disorders. The *CYP1A2* polymorphisms of −3860G>A, −2467delT or −739T>G were identified in these subjects, but they were always in linkage disequilibrium with other SNPs, e.g., with −163C>A. The CYP1A2*1F carrying −163C>A was found to be one of the rarest alleles, detected only in the psychiatric group with a frequency of 0.4%, which was significantly lower than the frequency of 32–57% reported previously in the literature [44]. This was attributed to the linkage disequilibrium of −163C>A with other *CYP1A2* SNPs, most frequently with 2159G>A in CYP1A2*1M. In the liver tissue donors and psychiatric patients, the most prevalent allele was CYP1A2*1M, at the frequencies of 59.9% and 62.6%, respectively, which was somewhat higher than what was observed by Djordjevic et al. in the Serbian population (54.8%) [25]. The CYP1A2*1L, CYP1A2*1V and CYP1A2*1W alleles were less common than CYP1A2*1M in both the investigated groups (CYP1A2*1L allele: 2% and 1.1%; CYP1A2*1V: 3.6% and 2.7%; and CYP1A2*1W: 1.7% and 1.1% in liver tissue donors and in psychiatric patients), similar to other Caucasian populations [20].

More than one tenth of the tissue donors and psychiatric patients carried none of the *CYP1A2* SNPs (12.6% and 12.8%, respectively) and were considered to be a CYP1A2*1/*1 carrier, whereas nearly 40% of the subjects were heterozygous CYP1A2*1/mut with the highest proportion of the CYP1A2*1/*1M genotype (35.8% of the liver tissue donors and 35% of the psychiatric patients). The CYP1A2*1/*1F genotype was identified only among the psychiatric patients in 2 of the 274 subjects. The proportions of CYP1A2*1/*1V (2% and 1.8%) and CYP1A2*1/*1W (1.3% and 1.1%) genotypes in the liver tissue donors and in the psychiatric patients, respectively, were similar. Individuals with CYP1A2*1/*1L genotype occurred only among the liver tissue donors (2 of 151 subjects). Nearly half of the subjects (47% of the liver tissue donors and 48.7% of psychiatric patients) were found to have a *CYP1A2mut/mut* genotype. The most common *CYP1A2mut/mut* genotype was CYP1A2*1M/*1M (37.1% of liver tissue donors and 42.7% of psychiatric patients), whereas the CYP1A2*1M/*1L, CYP1A2*1M/*1V, CYP1A2*1M/*1W, CYP1A2*1V/*1L, CYP1A2*1V/*1V and CYP1A2*1W/*1L genotypes were much less prevalent (Table 2).

### 3.2. Hepatic CYP1A2 Activities and mRNA Expression

**Figure 1:**
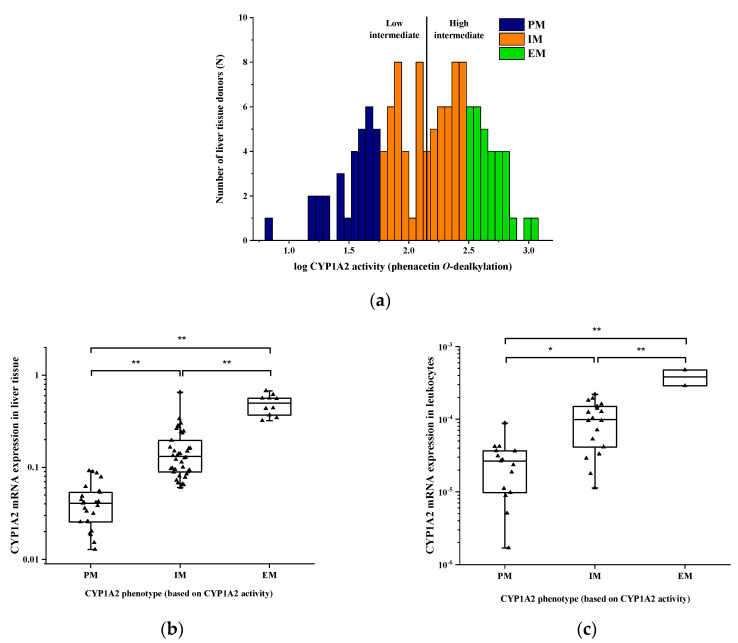
CYP1A2 activities and expression in liver tissue donors: (**a**) Frequency distribution of hepatic CYP1A2 activities (phenacetin *O*-dealkylation); (**b**) association between CYP1A2 activities and mRNA expression in the liver or (**c**) mRNA expression in the peripheral leukocytes of human tissue donors. Abbreviations: PM, poor metabolizer; IM, intermediate metabolizer; EM, normal metabolizer. The vertical line between low and high intermediate metabolizers indicates the median activity. In (**b**) and (**c**), the boxes display the range; the horizontal lines are for the median, and the whiskers are for the minimum-maximum values. * *p* < 0.01; ** *p* < 0.001.

For characterization of CYP1A2 enzyme activity, phenacetin was used as the CYP1A2-selective probe substrate in the microsomal fraction of 131 liver tissue samples. Phenacetin *O*-dealkylation ranged from extremely low to rather high values, which displayed a skewed distribution and more than two orders of magnitude differences between the lowest and the highest activities [6.76–1107.1 pmol/(mg ∗ min)] (Figure 1a). Based on the CYP1A2 activities, the liver tissue donors were classified into poor, intermediate and normal metabolizer phenotype categories, and the cutoff values between the categories were 60.2 and 281.7 pmol/(mg ∗ min), according to Temesvári et al. [45]. Of the liver tissues, 68 displayed intermediate CYP1A2 activity, while 31 of the 131 were poor and 32 were characterized as normal metabolizers. Significant association was found between the phenacetin *O*-dealkylation activity and CYP1A2 mRNA expression in the liver tissues (N = 73, *p* < 0.0001), and substantial differences in hepatic CYP1A2 mRNA expression were observed between poor, intermediate and normal metabolizers (Figure 1b). Furthermore, the differences in the CYP1A2 mRNA expression in leukocytes were also significant between the various CYP1A2 phenotype groups classified by hepatic CYP1A2 activity (N = 35, *p* < 0.0001) (Figure 1c).

### 3.3. Impact of −163C>A (rs762551) on CYP1A2 Activity and mRNA Expression in the Liver

**Figure 2:**
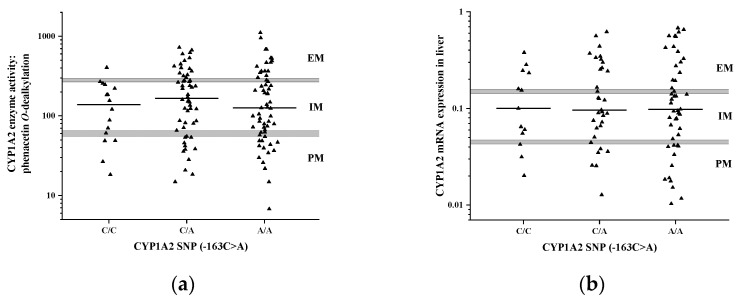
The association between −163C>A SNP (rs762551) in the regulatory region of *CYP1A2* gene and (**a**) hepatic CYP1A2 activity or (**b**) mRNA expression. The horizontal lines display the median values. Abbreviations: PM, poor metabolizer; IM, intermediate metabolizer; EM, normal metabolizer.

A higher CYP1A2 inducibility has been reported in subjects carrying −163A in the *CYP1A2* regulatory region than in those with C/C at −163 position; however, the impact of −163C>A failed to be demonstrated in some studies [25,26]. Of 131 liver tissue donors, 115 subjects carried -163A in heterozygous or homozygous form. Approximately half of the tissue donors (N = 61) were homozygous (-163A/A), 54 donors were heterozygous (−163C/A) and 16 were −163C/C carriers (Figure 2a). In liver tissue donors, −163C>A polymorphism appeared to have no influence on either the phenacetin *O*-dealkylation activity of CYP1A2 [−163C/C carriers: 150.2 ± 109.4 pmol/(mg ∗ min), −163C/A carriers: 222.1 ± 182.4 pmol/(mg ∗ min), −163A/A carriers: 216.9 ± 227.5 pmol/(mg ∗ min); N = 131, *p* = 0.425] (Figure 2a) or the hepatic CYP1A2 mRNA expression (Figure 2b). The proportion of the activity-based poor metabolizers was the same in the three −163C>A groups (22–25%), whereas normal metabolizers occurred more frequently in −163A carriers than in -163C/C subjects (6% in C/C carriers vs. 28% and 26% in C/A and in A/A carriers) (Figure 2a); the difference, however, was statistically not significant (N = 131, *p* = 0.1167). (See also the results of multivariate analysis in Section 3.4).

### 3.4. Impact of Genetic and Non-Genetic Factors on Inter-Individual Variation in Hepatic CYP1A2 Activities and Expression

**Figure 3:**
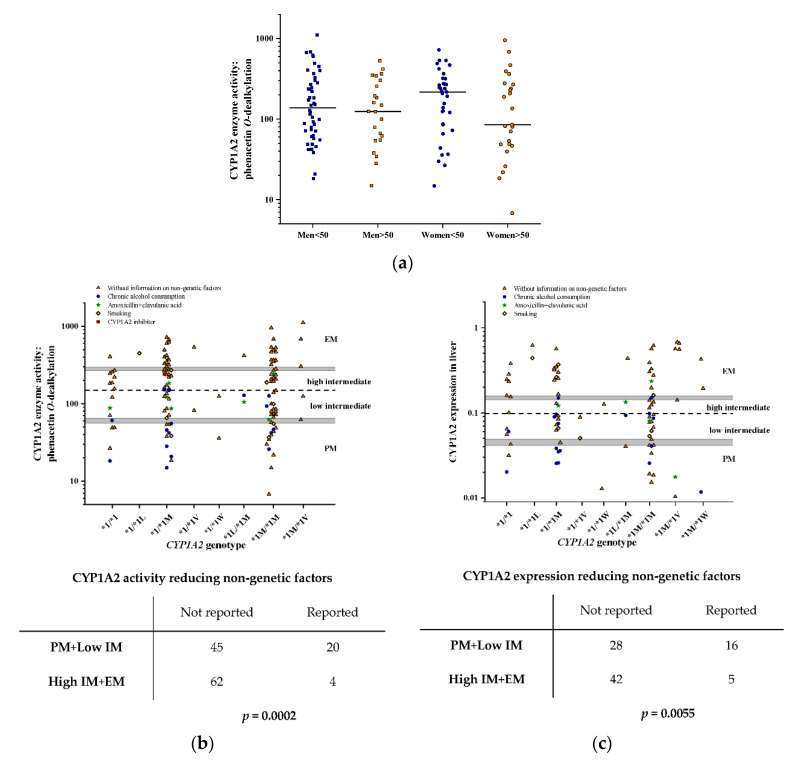
The effect of genetic and non-genetic factors on hepatic CYP1A2 activity (phenacetin *O*-dealkylation) and CYP1A2 expression in liver tissue donors: (**a**) The influence of gender and age of the subjects on CYP1A2 activity (<50 years old in blue, >50 years old in orange); (**b**) the impact of *CYP1A2* genetic variations and non-genetic factors (CYP1A2 inhibitor therapy, smoking, amoxicillin+clavulanic acid treatment, chronic alcohol consumption) on CYP1A2 activity and (**c**) on mRNA expression are presented. The median CYP1A2 activity (dotted line) is for the cutoff value between high and low intermediate metabolizers. Tables provide the number of subjects in various metabolizer groups with the information of relevant non-genetic factors. Abbreviations: PM, poor metabolizer; IM, intermediate metabolizer; EM, normal metabolizer.

Controversial data have been published about gender and age-related differences in CYP1A2 function [31,46]; therefore, the effect of sex and age on phenacetin *O*-dealkylation activity was investigated in the liver tissue donors. The CYP1A2 activity data were arranged into four groups: males and females as well as under 50 and over 50 years of age (Figure 3a). Only adult subjects (>18 years) were involved in the present study, and further subdivision by age would have been desirable; however, the group-size was unacceptably small. The CYP1A2 activity varied over two orders of magnitude in all four groups, and no significant differences were observed between males and females younger than 50 and older than 50 years old [male < 50 years: 220.6 ± 222.9 pmol/(mg ∗ min), male > 50 years: 175.3 ± 145.2 pmol/(mg ∗ min), females < 50 years: 228.0 ± 172.6 pmol/(mg ∗ min), females > 50 years: 201.2 ± 228.9 pmol/(mg ∗ min), *p* = 0.763].

Although no association was found between the −163C>A polymorphism and phenacetin *O*-dealkylation activity or CYP1A2 mRNA expression, other *CYP1A2* SNPs, including −3860G>A, −2467delT, −739T>G and 2159G>A, were also considered genetic determinants in CYP1A2 function. PharmVar has indicated several *CYP1A2* functional variants, to which decreased activity and expression (CYP1A2*1C, CYP1A2*1K) or increased inducibility (CYP1A2*1F) were attributed. In liver tissue donors, neither CYP1A2*1C, CYP1A2*1K, nor CYP1A2*1F were identified, because −3860G>A, −163C>A or −739T>G were always in linkage disequilibrium with other *CYP1A2* SNPs (Table 2). *CYP1A2* haplotype reconstruction identified CYP1A2*1L, CYP1A2*1M, CYP1A2*1V and CYP1A2*1W (Figure 3b,c), which have not been reported to be associated with altered CYP1A2 activity or expression. CYP1A2*1M, the most frequent allelic variant appeared to have no impact on hepatic phenacetin *O*-dealkylation activity either in heterozygous (CYP1A2*1/*1M) or in homozygous subjects (CYP1A2*1M/*1M) compared to CYP1A2*1/*1 carriers [CYP1A2*1/*1: 150.2 ± 109.4; CYP1A2*1/*1M: 219.8 ± 179.7; CYP1A2*1M/*1M: 194.3 ± 194.4 pmol/(mg ∗ min), N = 118, *p* = 0.393] (Figure 3b). Furthermore, no association between CYP1A2 mRNA expression and CYP1A2*1M was demonstrated (CYP1A2*1/*1: 0.141 ± 0.114; CYP1A2*1/*1M: 0.163 ± 0.138; CYP1A2*1M/*1M: 0.145 ± 0.149, N = 73, *p* = 0.851) (Figure 3c). In other *CYP1A2* genotype groups, the number of data was insufficient for statistical analysis.

In addition to the *CYP1A2* genetic variations, the impact of non-genetic factors on CYP1A2 activity and mRNA expression was also investigated (Figure 3b,c). The CYP1A2-inducing factor (smoking) and the CYP1A2 inhibitor ciprofloxacin, as well as non-specific non-genetic factors (chronic alcohol consumption and amoxicillin+clavulanic acid therapy) that can modify (increase or decrease) CYP1A2 activity and/or expression, were considered in the evaluation of CYP1A2 function [16,47]. Due to the incomplete smoking data in the clinical histories of the liver tissue donors, the CYP1A2-inducing effect of smoking was not evaluated in these subjects. However, smoking status was accurately reported for the patients with psychiatric disorders; therefore, the impact of this non-genetic factor was reliably assessed in this patient population. Although the 16 liver tissue donors with CYP1A2*1/*1 genotype were expected to be intermediate metabolizers, four displayed poor metabolism and one displayed normal metabolism. Among the poor metabolizers carrying CYP1A2*1/*1, two subjects reported chronic alcohol consumption, whereas one tissue donor, classified as low intermediate metabolizer, received amoxicillin+clavulanic acid therapy that likely explained the low phenacetin *O*-dealkylation. The CYP1A2 activity of the 49 organ donors carrying CYP1A2*1/*1M genotype ranged from poor to normal metabolism [14.85–724 pmol/(mg ∗ min)]. Chronic alcohol consumption (N = 7) and amoxicillin+clavulanic acid therapy (N = 2) as non-specific non-genetic factors explained the poor and low intermediate CYP1A2 activity in nine subjects, whereas the CYP1A2 inhibitor ciprofloxacin did not shift CYP1A2 activity into the poor metabolizer category. In the 53 tissue donors with CYP1A2*1M/*1M genotype, phenacetin *O*-dealkylation activity ranged from 6.76 pmol/(mg ∗ min), the lowest, to 950.2 pmol/(mg ∗ min), the highest. Similar to subjects with the CYP1A2*1/*1M genotype, chronic alcohol consumption (N = 5) and amoxicillin+clavulanic acid therapy (N = 1) in the clinical histories of the CYP1A2*1M/*1M carriers were associated with poor and low intermediate CYP1A2 activity. In the other *CYP1A2* genotype groups, either no information about non-genetic factors was available, or the non-specific non-genetic factors were found in the clinical histories (in CYP1A2*1L/*1M: one subject with chronic alcohol consumption and one with amoxicillin+clavulanic acid therapy), which explained the low intermediate activity. Taken together, non-specific non-genetic factors were assumed to be responsible for the poor and low intermediate CYP1A2 activity in 20 of 65 tissue donors (chronic alcohol consumption: 15; amoxicillin+clavulanic acid therapy: five), whereas in those subjects with high intermediate and normal metabolism (N = 66), non-genetic factors were identified in the clinical histories of four tissue donors (chronic alcohol consumption: one; amoxicillin+clavulanic acid therapy: two; ciprofloxacin: one). In other words, the CYP1A2 activity-reducing non-genetic factors were reported more frequently in the poor and low intermediate metabolizer subjects than in the high intermediate and normal metabolizers (20/65 vs. 4/66; OR: 6.889, 95%CI: 2.202–21.548, *p* = 0.0002) (Figure 3b).

The impact of non-specific non-genetic factors (chronic alcohol consumption, amoxicillin+clavulanic acid therapy) on CYP1A2 mRNA expression in the liver was established in 93 liver tissue donor subjects (Figure 3c). Ciprofloxacin has been demonstrated to exert an inhibitory effect on the CYP1A2 enzyme protein [44,48]; however, it was not considered a non-genetic factor that can modify CYP1A2 expression. Hepatic CYP1A2 mRNA expression was influenced by non-specific non-genetic factors similar to phenacetin *O*-dealkylation activity. Chronic alcohol consumption (N = 16) and amoxicillin+clavulanic acid therapy (N = 2) were likely to be responsible for the poor and low intermediate CYP1A2 expression in 18 of 46 tissue donors, whereas these non-genetic factors were found in the clinical histories of five high intermediate and normal metabolizers. Non-specific non-genetic factors were more frequently reported in those subjects with reduced CYP1A2 expression than in the high intermediate and normal expressers (18/46 vs. 5/47; OR: 5.4, 95%CI: 1.797–16.23, *p* = 0.0017) (Figure 3c).

A multiple linear regression analysis was also performed to estimate the influence of genetic (*CYP1A2* SNPs or haplotypes) and non-genetic covariates (sex, age, medication with amoxicillin+clavulanic acid and chronic alcohol consumption) on CYP1A2 activity and on CYP1A2 mRNA expression (Table 3). Significant associations were observed between phenacetin *O*-dealkylation activity and nucleotide deletion −2467delT (*p* = 0.011), age (*p* = 0.027) or chronic alcohol consumption (*p* < 0.001). When the *CYP1A2* haplotypes were considered, the impact of the −3860G/−2467delT/−739T/−163A/2159G haplotype, designated as CYP1A2*1V, was significant (*p* = 0.004), and age (*p* = 0.024) and CYP1A2 activity-reducing non-genetic factors (chronic alcohol consumption and amoxicillin+clavulanic acid therapy) appeared to be associated with phenacetin *O*-dealkylation activity (*p* < 0.001). Furthermore, hepatic CYP1A2 mRNA expression was significantly associated with the −2467delT (*p* = 0.011), amoxicillin+clavulanic acid therapy (*p* = 0.050) or chronic alcohol consumption (*p* = 0.004), but not with the age (*p* = 0.128). Involving *CYP1A2* haplotypes in the multiple regression model, the association between CYP1A2 mRNA expression and the −3860A/−2467delT/−739T/−163A/2159G and −3860G/−2467delT/−739T/−163A/2159G haplotypes, present in CYP1A2*1L and CYP1A2*1V alleles, became significant (*p* = 0.019 and *p* = 0.009, respectively). The CYP1A2 expression that reduces non-genetic factors displayed significant association with hepatic CYP1A2 expression (*p* = 0.002). However, sex appeared to have no influence on either the CYP1A2 activity or mRNA expression.

### 3.5. Impact of Genetic and Non-Genetic Factors on CYP1A2 mRNA Expression in Psychiatric Patients

**Figure 4:**
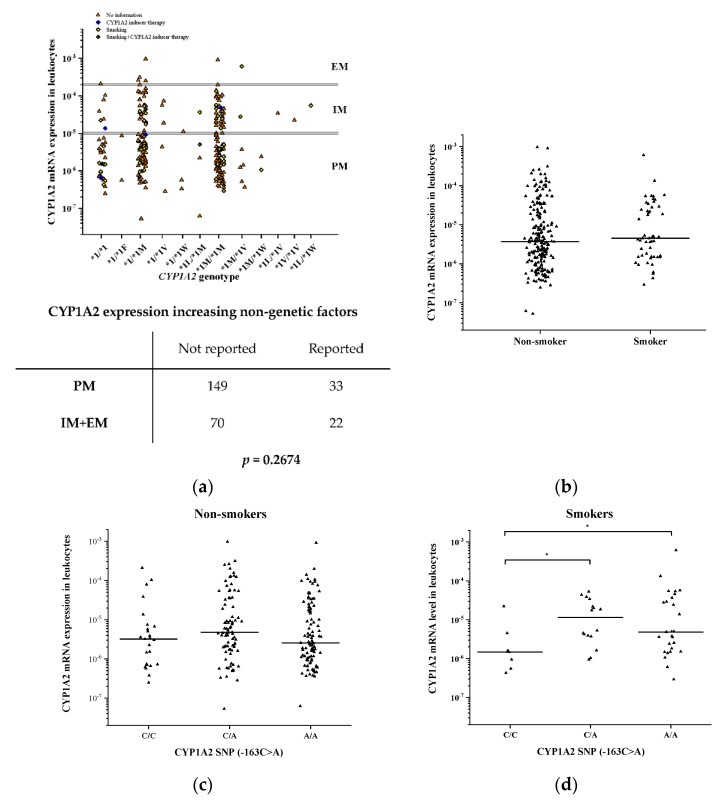
The effect of genetic and non-genetic factors on CYP1A2 expression in the leukocytes of patients with psychiatric disorders: The impact of *CYP1A2* genetic variations and non-genetic factors (CYP1A2-inducer therapy, smoking) on CYP1A2 expression (**a**) and the influence of smoking on mRNA expression (**b**–**d**) are presented. The horizontal lines display the median values. The table provides the number of subjects in various metabolizer groups with the information of relevant non-genetic factors. Abbreviations: PM, poor metabolizer; IM, intermediate metabolizer; EM, normal metabolizer; * *p* < 0.05.

The *CYP1A2* genotype of the patients with psychiatric disorders was determined from *CYP1A2* SNP data (including −3860G>A, −2467delT, −739T>G, −163C>A and 2159G>A) by haplotype analysis, whereas the hepatic CYP1A2 function was estimated from CYP1A2 mRNA expression in patients’ leukocytes. Four orders of magnitude difference in the CYP1A2 expression were observed between the lowest and the highest mRNA levels (Figure 4a). Approximately two thirds of the patients were classified as CYP1A2 poor metabolizers, 31% as intermediate and only 2.6% as normal metabolizers. In psychiatric patients, no association was found between the *CYP1A2* genotypes and CYP1A2 mRNA expression in leukocytes (N = 274, *p* = 0.1165) (Figure 4a).

The polycyclic aromatic hydrocarbons in tobacco smoke have been demonstrated to increase CYP1A2 activity by increasing the CYP1A2 expression via the AhR-mediated signalization pathway [49]. In the liver tissue donors, the contribution of smoking as a CYP1A2-inducing factor to CYP1A2 expression was not evaluated because of incomplete information about their smoking status. However, smoking habits and CYP1A2-inducer therapy (with carbamazepine) were well documented in the group of patients with psychiatric disorders; therefore, the impact of these non-genetic phenoconverting factors on the CYP1A2 expression was reliably investigated (Figure 4). Smoking and CYP1A2-inducer carbamazepine therapy were reported in most of the *CYP1A2* genotype groups and in both poor and intermediate/normal metabolizers. However, no significant differences in the frequency of the CYP1A2-inducing factors in the clinical histories were observed between the poor and intermediate/normal metabolizers (33/182 vs. 22/92, *p* = 0.2674). Furthermore, smoking seemed to have no influence on CYP1A2 expression when *CYP1A2* genetic factors were not considered (non-smokers: 3.23 × 10^−5^ ± 9.99 × 10^−5^; smokers: 2.82 × 10^−5^ ± 8.65 × 10^−5^, *p* = 0.7858) (Figure 4b).

According to several previous studies, the CYP1A2-inducing effect of tobacco smoking is more prominent in subjects with -163C>A SNP in the *CYP1A2*-regulatory region than in those with −163C/C [20,50]; therefore, we evaluated the effect of -163A on CYP1A2 inducibility, both in smokers and in non-smoker patients. Those patients who were on carbamazepine therapy were excluded from the evaluation (N = 6). In the non-smokers, no significant differences in the CYP1A2 mRNA level were observed between various *CYP1A2* −163C>A carriers (−163C/C: 2.02 × 10^−5^ ± 4.67 × 10^−5^; −163C/A: 4.31 × 10^−5^ ± 1.16 × 10^−4^; −163A/A: 2.59 × 10^−5^ ± 9.36 × 10^−5^, N = 268; *p* = 0.4028) (Figure 4c). Of the 52 smoker subjects, the wild-type −163C/C carriers had significantly lower CYP1A2 mRNA expression in leukocytes compared to patients with −163C/A and −163A/A (−163C/C: 4.17 × 10^−6^ ± 7.45 × 10^−6^; −163C/A: 1.71 × 10^−5^ ± 1.71 × 10^−5^; −163A/A: 4.20 × 10^−5^ ± 1.16 × 10^−4^, N = 52, *p* = 0.0369) (Figure 4d).

## 4. Discussion

The present work investigated the association between *CYP1A2* genetic polymorphisms and CYP1A2-selective phenacetin *O*-dealkylation activity or CYP1A2 mRNA expression in human liver tissues and in psychiatric patients belonging to Caucasian populations. A high inter-individual variability in the CYP1A2 activity and expression was observed in the liver tissue donors, which was consistent with the results obtained from human liver samples in a recent study by Liu et al. [31] and with the CYP1A2 phenotype prediction in patients by Lorenzini et al. [51]. The frequencies of the liver tissue donors with poor, intermediate and normal CYP1A2 activities were more or less similar to the frequency distribution of poor, normal and ultra-rapid metabolizer phenotypes in patients reported by Lorenzini et al. [51]. In a previous study [52], a strong correlation was reported between hepatic CYP1A2 activity and mRNA expression, which was confirmed by our findings in the liver tissue donors. Furthermore, a significant association between the hepatic CYP1A2 enzyme activity and mRNA expression in leukocytes was observed, similar to the previous findings by Temesvári et al. [45]; thus, CYP1A2 mRNA expression in leukocytes appeared to be an appropriate, less-invasive marker for the estimation of hepatic CYP1A2 activity in patients.

The substantial variability in hepatic CYP1A2 function was attributed to both genetic polymorphisms and non-genetic factors [10,27,51]. Several *CYP1A2* allelic variants have been associated with altered CYP1A2 activity and/or mRNA expression. CYP1A2*1F has been reported to be one of the most common allelic variants, with 32-57% allele frequencies in Caucasian populations [20,44,53]; however, due to the close genetic linkage between −163C>A SNP and other SNPs (e.g., −3860G>A, −2467delT, −739T>G or 2159G>A), generating haplotypes other than CYP1A2*1F, the frequency data should be applied to −163A rather than to CYP1A2*1F [54]. When involving the most common SNPs in the haplotype reconstruction, we found CYP1A2*1F to be one of the rarest *CYP1A2* haplotypes, with a frequency of 0.4% in the subjects of the present study. Although the extremely low frequency of CYP1A2*1F did not allow for an association analysis with CYP1A2 activity and expression, the most frequent −163C>A SNP (67%) was found to have no influence on the hepatic phenacetin *O*-dealkylation activity or on the CYP1A2 mRNA expression. The −163C>A SNP has been suggested to be associated with increased inducibility; however, the contribution of −163C>A to increased CYP1A2 activity is inconsistent in the literature [15,16,17,18,19,20,21]. Increased caffeine metabolism (high paraxanthine/caffeine ratio) was demonstrated only in smokers carrying −163A/A, compared to those carrying −163C/C or −163C/A [17,20]. Furthermore, significantly lower olanzapine serum concentrations were observed in smokers with −163A/A, compared to those observed in the heterozygous or homozygous wild-type subjects [19]. Our findings confirmed the results of these previous studies; particularly the finding that elevated CYP1A2 mRNA expression was found in the smoker psychiatric patients carrying two, or even one, −163A (−163A/A or 163C/A), compared to those with the wild-type −163C/C. However, no association between CYP1A2 expression and −163C>A polymorphism was observed in non-smoker patients. Dobrinas et al. have reported high paraxanthine/caffeine ratios in subjects carrying −163A, regardless of smoking [16]; however, in the liver tissue donors with incomplete smoking information in the present study, no association was found between −163C>A polymorphism and either phenacetin *O*-dealkylation activity or CYP1A2 mRNA expression. Decreased CYP1A2 activity has been attributed to CYP1A2*1C (−3860G>A) [16,20,24], whereas controversial results of CYP1A2 inducibility have been reported in CYP1A2*1D (−2467delT)-carrier smokers [16,18,20,55,56]. In the liver tissue donors and patients with psychiatric disorders involved in the present study, the −3860G>A and −2467delT SNPs were identified with frequencies similar to other European populations [20,55]; however, these SNPs were in linkage disequilibrium with −163C>A in CYP1A2*1L, CYP1A2*1V and CYP1A2*1W. The −3860G>A SNP was found to have no impact on CYP1A2 function, whereas the −2467delT SNP and CYP1A2*1V allele appeared to contribute to increased CYP1A2 activity and mRNA expression in liver tissues. Although the CYP1A2*1L-carrier subjects also displayed increased CYP1A2 mRNA expression, phenacetin *O*-dealkylation activity was not influenced by the CYP1A2*1L allele. The CYP1A2*1M allelic variant most common in Caucasian populations [25] occurred in approximately 60% of tissue donors and psychiatric patients and appeared to have no significant effect on CYP1A2 activity and expression.

For several CYPs and other drug-metabolizing enzymes, pharmacogenetic testing has been considered a useful tool for identification of high-risk patients with loss-of-function or gain-of-function alleles, and plenty of pharmacogenetic guidelines recommend dose adjustment of a drug to the patients’ genetic information [57,58,59,60]. For CYP1A2, no pharmacogenetic guideline has been published yet, most likely because several common *CYP1A2* SNPs are in the regulatory elements of the promoter region or in intron 1, and the complex regulatory mechanisms require the action of non-genetic phenoconverting factors. In the present study, we have demonstrated a minor association between SNPs in the regulatory regions of the *CYP1A2* gene or *CYP1A2* haplotypes and CYP1A2 activity or expression either in liver tissue donors or in psychiatric patients. Our results are in concordance with the conclusion of a recent clinical study [61] which found that *CYP1A2* genotyping has no, or minor clinical relevance in treatment with CYP1A2 substrate drugs. Several non-genetic intrinsic and environmental factors, such as age, sex, hormones, diseases, alcohol consumption and medication have been reported to modify CYP1A2 activity and expression [10,27,62]. Men have been demonstrated to have significantly higher microsomal CYP1A2 activity than women [29], whereas a significant decline in CYP1A2 activity was observed with age [46]. Although our results confirmed the decreased CYP1A2 activity in the subjects older than 50 years of age, no significant association between sexes and hepatic CYP1A2 activity or expression was found, similar to a recent study by Liu et al. [31]. Several clinical studies demonstrated that oral contraceptives contributed to decreased CYP1A2 activity by influencing hormonal status [36,62]. In the clinical histories of the liver tissue donors, treatment with oral contraceptives was not reported; however, chronic alcohol consumption or amoxicillin+clavulanic acid therapy as non-specific non-genetic factors that significantly decreased the CYP1A2 activity and expression were indicated. A large body of evidence has been provided to demonstrate that the pathomechanism of liver disease induced by chronic alcohol consumption (inflammation, oxidative stress) was related to impaired drug metabolism [63,64,65]. The antibiotic amoxicillin, in combination with clavulanic acid, has been reported to lead rarely to drug-induced liver injury or severe acute liver failure [66,67]. Although no information is available about the CYP1A2-selective inhibitory effect or downregulation of the CYP1A2 expression by chronic alcohol consumption or amoxicillin+clavulanic acid therapy, hepatotoxicity-related inflammation might have influenced the CYP1A2 function and/or expression. We have previously demonstrated that these non-specific non-genetic factors were mainly responsible for poor and low intermediate metabolism by CYP2C9 and CYP2C19 [68,69]. In addition to non-specific chronic alcohol consumption and amoxicillin+clavulanic acid therapy, CYP1A2-specific inducer and inhibitor therapy has clearly demonstrated contribution to the inter-individual variability in CYP1A2 activity and to be the major causes of phenoconversion of genetically determined CYP1A2 activities [35]. Co-administration of ciprofloxacin, the potent CYP1A2 inhibitor, has been found to significantly elevate the serum concentrations and exposure to CYP1A2 substrates [47]. For one liver tissue donor, ciprofloxacin therapy was indicated in the clinical history; however, inhibition of the hepatic phenacetin *O*-dealkylation was not observed. The inducibility of the *CYP1A2* gene via the AhR-mediated signaling pathway is well documented, and exposure to CYP1A2-specific inducing factors (carbamazepine, components of cigarette smoke) is logically expected to increase transcriptional expression of CYP1A2. Clinical studies have shown that smoking increased the clearance and reduced the plasma concentrations of CYP1A2 substrate drugs, such as clozapine or olanzapine [70,71]. Most of the studies reported 1.1-2-fold differences in CYP1A2 activity between smokers and non-smokers [44]. In contrast, no significant differences in CYP1A2 expression were observed between smoker and non-smoker patients with psychiatric disorders, when we did not consider genetic factors. However, the CYP1A2 inducing effect of smoking was demonstrated in the -163A carrier patients. Furthermore, treatment with the CYP1A2 inducer carbamazepine has been reported to significantly reduce the serum concentration of the CYP1A2 substrate olanzapine [19,72]. In the patients with psychiatric disorders, only six were on carbamazepine therapy, three displayed intermediate CYP1A2 expression and three were CYP1A2 poor metabolizers. Due to the low number of patients on carbamazepine therapy and the wide range of CYP1A2 expression, we could not confirm the CYP1A2-inducing effect of carbamazepine.

The present study has some limitations that should be discussed. First, we did not evaluate the effect of *CYP1A2* SNPs other than −3860G>A (rs2069514), −2467delT (rs35694136), –739T>G (rs2069526), −163C>A (rs762551) or 2159G>A (rs2472304), which are present in the CYP1A2*1C, CYP1A2*1D, CYP1A2*1E, CYP1A2*1F, CYP1A2*1L, CYP1A2*1M, CYP1A2*1V or CYP1A2*1W alleles. However, other *CYP1A2* alleles associated with altered CYP1A2 activity or expression (e.g., CYP1A2*3, CYP1A2*4, CYP1A2*6, CYP1A2*7, CYP1A2*8, CYP1A2*11 and CYP1A2*15) have not been reported or have been detected at a low prevalence in Caucasian populations [43,73]. Second, CYP1A2*1F was not identified in the liver tissue donors and was detected at extremely low frequency (0.4%) in the patients with psychiatric disorders; therefore, the effect of CYP1A2*1F on CYP1A2 activity or expression was not assessed. Third, no information about oral contraceptive therapy and incomplete information about smoking status was available in the clinical histories of the liver tissue donors. Although the impact of smoking on CYP1A2 expression was reliably evaluated in the patients with psychiatric disorders, oral contraceptives were not applied in these patients; therefore, no conclusion on the CYP1A2 activity-reducing effect of these drugs could be drawn.

## 5. Conclusions

The *CYP1A2* alleles, including CYP1A2*1C, CYP1A2*1D, CYP1A2*1F, CYP1A2*1L, CYP1A2*1M, CYP1A2*1V and CYP1A2*1W, were reconstructed from −3860G>A, −2467delT, –739T>G, −163C>A or 2159G>A SNP data in liver tissue donors and in patients with psychiatric disorders. CYP1A2*1F, generally identified by the presence of −163C>A polymorphism, has been considered one of the most common *CYP1A2* alleles (32–57%); however, due to linkage disequilibrium of −163C>A with other *CYP1A2* SNPs, the CYP1A2*1F allele was identified at an extremely low frequency (0–0.4%) in the subjects of the present study. A high inter-individual variability in the phenacetin *O*-dealkylation activity and CYP1A2 mRNA expression was detected in the liver tissue donors; however, the *CYP1A2* genetic polymorphisms appeared to have a minor impact on the hepatic CYP1A2 activity and expression. Non-genetic factors, such as smoking, chronic alcohol consumption and amoxicillin+clavulanic acid therapy, were found to influence CYP1A2 expression and activity. Smoking, the CYP1A2-inducing factor, contributed to increased CYP1A2 expression in −163A carrier patients, whereas the non-specific non-genetic factors, chronic alcohol consumption and amoxicillin+clavulanic acid treatment, were associated with decreased CYP1A2 function and expression. Although CYP polymorphism analysis generally provides significant information in the prediction of CYP-mediated metabolism, for CYP1A2, non-genetic factors, rather than genetic polymorphisms, appeared to contribute to CYP1A2 function. Furthermore, a strong association was demonstrated between the hepatic CYP1A2 activity and CYP1A2 mRNA expression in the liver or in the peripheral leukocytes. Therefore, an analysis of CYP1A2 mRNA expression in leukocytes, rather than *CYP1A2* genetic variants, may provide an appropriate tool for accurate prediction of hepatic CYP1A2 function and for the estimation of patients’ CYP1A2-metabolizing capacity toward drugs, such as clozapine, olanzapine, duloxetine, theophylline or tacrine.

## Figures and Tables

**Table 1:** Clinical history of the human subjects.

	Liver Tissue Donors		Psychiatric Patients	
Number of subjects	151		274	
Age (year) ^1^	46 (18; 74)		39 (18; 76)	
Gender (male/female)	82/69		110/164	
Cause of death			Psychiatric disorders	
Accident	Car/motor/bike accident	14	Schizophrenia	140
	Anoxic cerebral injury/asphyxia	5	Persistent delusional disorders	2
	Seizure induced cerebral injury	3	Acute and transient psychotic disorders	16
	Suicide	4	Schizoaffective disorders	44
	Unknown cerebral injury	9	Other non-organic psychotic disorders	2
Cerebral hemorrhage/hematoma	Ruptured cerebral aneurysm	4	Bipolar affective disorder	52
	Epidural hematoma	1	Depressive episode	5
	Intraventricular hemorrhage	8	Recurrent depressive disorder	3
	Subarachnoid hemorrhage	31	Other anxiety disorders	1
	Subdural hemorrhage	8	Specific personality disorders	2
	Unknown cerebral hemorrhage	15	Unknown	7
Stroke	Ischemic stroke	7		
	Hemorrhagic stroke	2		
Tumor		36		
Unknown		4		
Non-genetic factors				
Amoxicillin+clavulanic acid therapy	8		0	
Chronic alcohol consumption	19		0	
Medication with CYP1A2 inducer	0		6	
Medication with CYP1A2 inhibitor	1		0	
Smoking	8		96	

^1^ median (min; max).

**Table 2:** *CYP1A2* allele and genotype frequencies in the tissue donors, psychiatric patients and in Caucasian populations.

*CYP1A2* Allele	Nucleotide Changes	N		Frequency (%)		
		Tissue donors	Psychiatric patients	Tissue donors	Psychiatric patients	Caucasian populations ^1^
*1 ^2^	None	99	176	32.8	32.1	24.4–63.5
*1C	−3860G>A	0	0	0	0	0.4–4
*1D	−2467delT	0	0	0	0	3.4–11
*1E	−739T>G	0	0	0	0	0.4–6
*1F	−163C>A	0	2	0	0.4	32–57
*1L	−3860G>A; −2467delT; −163C>A; 5347T>C	6	6	2.0	1.1	0.8
*1M	−163C>A; 2159G>A	181	343	59.9	62.6	54.8
*1V	−2467delT; −163C>A	11	15	3.6	2.7	2.8–12.3
*1W	−3113A>G; −2467delT; −739T>G; −163C>A	5	6	1.7	1.1	1.2–2.1
*CYP1A2* genotype	N			Frequency (%)		
	Tissue donors	Psychiatric patients		Tissue donors	Psychiatric patients	
*1/*1	19	35		12.6	12.8	
*1/*1F	0	2		0	0.7	
*1/*1L	2	0		1.3	0	
*1/*1M	54	96		35.8	35.0	
*1/*1V	3	5		2.0	1.8	
*1/*1W	2	3		1.3	1.1	
*1M/*1L	4	4		2.6	1.5	
*1M/*1M	56	117		37.1	42.7	
*1M/*1V	8	7		5.3	2.6	
*1M/*1W	3	2		2	0.7	
*1V/*1L	0	1		0	0.4	
*1V/*1V	0	1		0	0.4	
*1W/*1L	0	1		0	0.4	

^1^ allele frequencies in Caucasian populations according to [20,25,33,43]. ^2^ The CYP1A2*1 wild-type allele is often designated as CYP1A2*1A.

**Table 3:** Multivariate analysis of CYP1A2 activity (phenacetin *O*-dealkylation) and mRNA expression, considering genetic (*CYP1A2* SNPs or haplotypes) and non-genetic factors in liver tissue donors.

Variable		CYP1A2 Activity			CYP1A2 mRNA Expression		
		Coefficient B (SE)	Coefficient ß	*p* Value	Coefficient B (SE)	Coefficient ß	*p* Value
**SNPs, non-genetic**	Constant	124.92 (54.99)		0.025	0.125 (0.052)		0.017
	−3860G>A (rs2069514)	−87.27 (119.26)	−0.077	0.466	0.014 (0.086)	0.021	0.867
	−163C>A (rs762551)	−2.77 (136.61)	−0.005	0.984	−0.034 (0.098)	−0.070	0.728
	−2467delT (rs35694136)	212.01 (81.90)	0.312	**0.011**	0.164 (0.063)	0.385	**0.011**
	−739T>G (rs20695)	−288.73 (218.63)	−0.128	0.189	−0.130 (0.100)	−0.154	0.198
	2159G>A (rs2472304)	70.81 (127.58)	0.130	0.580	0.084 (0.088)	0.194	0.341
	Sex	−8.02 (34.82)	−0.020	0.818	−0.023 (0.040)	−0.066	0.569
	Age	78.69 (35.06)	0.191	**0.027**	0.058 (0.038)	0.166	0.128
	Amoxicillin+clavulanic acid therapy	−133.89 (74.94)	−0.154	0.077	−0.161 (0.081)	−0.213	**0.050**
	Chronic alcohol consumption	−170.67 (50.01)	−0.292	**<0.001**	−0.146 (0.049)	−0.338	**0.004**
**Haplotypes, non-genetic**	Constant	124.12 (51.83)		0.018	0.119 (0.046)		0.011
	**−3860A**/**−2467delT**/−739T/**−163A**/2159G	127.91 (95.67)	0.112	0.184	0.166 (0.069)	0.238	**0.019**
	−3860G/−2467T/−739T/**−163A**/**2159A**	68.27 (46.56)	0.125	0.145	0.057 (0.044)	0.132	0.199
	−3860G/**−2467delT**/−739T/**−163A**/2159G	211.41 (72.86)	0.243	**0.004**	0.154 (0.058)	0.267	**0.009**
	−3860G/**−2467delT**/**−739G**/**−163A**/2159G	−79.18 (190.15)	−0.035	0.678	0.027 (0.082)	0.032	0.743
	Sex	−7.59 (34.35)	−0.019	0.825	−0.021 (0.039)	−0.060	0.592
	Age	79.19 (34.76)	0.193	**0.024**	0.057 (0.037)	0.163	0.127
	Activity reducing factors	−160.33 (43.82)	−0.316	**<0.001**	−0.149 (0.045)	−0.375	**0.002**

In haplotypes, the polymorphic variants were indicated in bold. The *p* values < 0.05 were considered to be statistically significant and are indicated in bold.

## Data Availability

Data are contained within the article.

## Supplementary Materials

The following supporting information can be downloaded at: https://www.mdpi.com/article/10.3390/pharmaceutics14030532/s1. Table S1: Sequences of primers and fluorophore-labelled probe oligonucleotides for real-time PCR assaying *CYP1A2* SNPs. Table S2: The flowchart of data analysis obtained from liver tissues.
