# Supplementary material for: CYP1A2 mRNA Expression Rather than Genetic Variants Indicate Hepatic CYP1A2 Activity
Source: Pharmaceutics. 2022 Feb 27;14(3):532. doi: 10.3390/pharmaceutics14030532 (PMC8954692; doi:10.3390/pharmaceutics14030532)
Supplement: Supplementary file 1 [file pharmaceutics-14-00532-s001.zip › pharmaceutics-1577577-supplementary.pdf]

pharmaceutics

Supplementary Materials: CYP1A2 mRNA Expression rather than Genetic Variants Indicate Hepatic CYP1A2 Activity

Ferenc Fekete, Katalin Mangó, Annamária Minus, Katalin Tóth and Katalin Monostory

Table S1. Sequences of primers and fluorophore-labelled probe oligonucleotides for real-time PCR assaying *CYP1A2* SNPs.

SNP		Sequence (5′-3′)
**-163C>A (rs762551)**	Forward primer	AGAGAGCCAGCGTTCATG
	Reverse primer	TGATGCGTGTTCTGTGCT
	Wild-type probe	FAM-CATGCGTCCTG**G**GCCCACAG-BHQ1
	Mutant probe	FAM-CATGCGTCCTG**T**GCCCACAGG-BHQ2
-3860G>A (rs2069514)	Forward primer	ATCAAGCTACACATGATCG
	Reverse primer	GGTGCACACCTGTAATTC
	Wild-type probe	FAM-CCTCTC**G**GATTCAAGCAATTGTCATGC-BHQ1
	Mutant probe	HEX-CCTCTC**A**GATTCAAGCAATTGTCATGC-BHQ2
2159G>A (rs2472304)	Forward primer	GATCCAGAAGGAGCTGGG
	Reverse primer	CACACAGCAGGCACATAAC
	Wild-type probe	FAM-CAGGAGAAGCCTTGA**G**ACCCAGG-BHQ1
	Mutant probe	HEX-CAGGAGAAGCCTTGA**A**ACCCAGG-BHQ2

FAM, HEX fluorophores, BHQ1, BHQ2 quenchers.

Table S2. The flowchart of data analysis obtained from liver tissues.

**Association of CYP1A2 function (activity or mRNA expression) with genetic and non-genetic variables** (multiple linear regression analysis)		
	Models 1 and 2	Models 3 and 4
Independent variables	Dependent variables	Dependent variables
CYP1A2 SNPs and non-genetic factors (sex, age, function reducing factors [a], function increasing factors[b])	→CYP1A2 activity in the liver	→CYP1A2 mRNA in the liver
CYP1A2 haplotypes and non-genetic factors (sex, age, function reducing factors[a], function increasing factors [b])	→CYP1A2 activity in the liver	→CYP1A2 mRNA in the liver
**Association between CYP1A2 activity and mRNA expression** (linear regression analysis)		
CYP1A2 mRNA in the liver	→CYP1A2 activity in the liver	
CYP1A2 mRNA in leukocytes	→CYP1A2 activity in the liver	

[a] CYP1A2 inhibitor (ciprofloxacin), amoxicillin+clavulanic acid therapy, chronic alcohol consumption; [b] CYP1A2 inducer.
